# Serial femtosecond crystallography of soluble proteins in lipidic cubic phase

**DOI:** 10.1107/S2052252515013160

**Published:** 2015-08-04

**Authors:** Raimund Fromme, Andrii Ishchenko, Markus Metz, Shatabdi Roy Chowdhury, Shibom Basu, Sébastien Boutet, Petra Fromme, Thomas A. White, Anton Barty, John C. H. Spence, Uwe Weierstall, Wei Liu, Vadim Cherezov

**Affiliations:** a Center for Applied Structural Discovery at the Biodesign Institute, Department of Chemistry and Biochemistry, Arizona State University, 727 East Tyler Street, Tempe, AZ 85287, USA; b Bridge Institute, Department of Chemistry, University of Southern California, 3430 South Vermont Avenue, MC 3303, Los Angeles, CA 90089, USA; c Center for Free Electron Laser Science, Deutsches Elektronen-Synchrotron (DESY), Notkestrasse 85, 22607 Hamburg, Germany; d Department of Physics, University of Hamburg, Luruper Chaussee 149, 22761 Hamburg, Germany; e Linac Coherent Light Source, SLAC National Accelerator Laboratory, 2575 Sand Hill Road, Menlo Park, CA 94025, USA; f Department of Physics, Arizona State University, PO Box 871504, Tempe, AZ 85287, USA

**Keywords:** serial femtosecond crystallography, X-ray free-electron laser, lipidic cubic phase, soluble protein

## Abstract

Serial femtosecond crystallography (SFX) at X-ray free-electron lasers (XFELs) enables high-resolution protein structure determination using micrometre-sized crystals at room temperature with minimal effects from radiation damage. SFX requires a steady supply of microcrystals intersecting the XFEL beam at random orientations. An LCP–SFX method has recently been introduced in which microcrystals of membrane proteins are grown and delivered for SFX data collection inside a gel-like membrane-mimetic matrix, known as lipidic cubic phase (LCP), using a special LCP microextrusion injector. Here, it is demonstrated that LCP can also be used as a suitable carrier medium for microcrystals of soluble proteins, enabling a dramatic reduction in the amount of crystallized protein required for data collection compared with crystals delivered by liquid injectors. High-quality LCP–SFX data sets were collected for two soluble proteins, lysozyme and phycocyanin, using less than 0.1 mg of each protein.

## Abstract 2

A new approach of using lipidic cubic phase as a carrier matrix for delivering soluble protein microcrystals for serial crystallography helps to dramatically reduce protein consumption. The structures of two soluble test proteins have been determined by this method using less than 0.1 mg of each protein.

## 1. Introduction   

The recent advent of hard X-ray free-electron lasers (XFELs) has opened up many exciting opportunities in structural biology (Feld & Frank, 2014 ▸). Intense XFEL pulses of extreme­ly short duration (<50 fs) make it possible to outrun radiation damage, as predicted by Neutze *et al.* (2000 ▸) and as later demonstrated by Chapman *et al.* (2006 ▸), enabling structure determination from tiny crystals at room temperature. Data are usually acquired using a serial femtosecond crystallography (SFX) approach, in which a continuous stream of microcrystals is intersected with the XFEL beam and diffraction patterns from individual crystals at random orientations are recorded at the pulse repetition rate of the laser (Chapman *et al.*, 2011 ▸). Following the automated indexing and merging of thousands of diffraction patterns, a Monte Carlo method is used to integrate over the angular profile of the Bragg reflections and other stochastic fluctuations (Kirian *et al.*, 2011 ▸). SFX has been successfully applied to both soluble proteins (Boutet *et al.*, 2012 ▸; Redecke *et al.*, 2013 ▸; Sawaya *et al.*, 2014 ▸) and membrane proteins (Johansson *et al.*, 2012 ▸; Kern *et al.*, 2013 ▸; Johansson *et al.*, 2013 ▸) using crystals ranging in size from nanometres to micrometres. These were formed in either aqueous solution or lipidic sponge phase and continuously delivered to the XFEL beam through a fast-running liquid microjet produced by a gas dynamic virtual nozzle (GDVN) injector (DePonte *et al.*, 2008 ▸). Initial proof-of-concept experiments have shown the potential of this technology for structural studies of proteins that are refractory to the growth of sufficiently large, high-quality crystals suitable for data collection at synchrotron sources, as well as for time-resolved studies of unstable intermediate states and irreversible processes. This technique, however, requires very large amounts (10–100 mg) of crystalline material for data collection, most of which runs to waste between shots, limiting its use to only well expressed and well behaved proteins. To address this issue, we have previously introduced an LCP–SFX method (Liu *et al.*, 2013 ▸, 2014 ▸; Weierstall *et al.*, 2014 ▸) in which membrane proteins are crystallized and delivered for data collection inside a gel-like lipidic cubic phase (LCP). The specific texture and high viscosity of the LCP medium allows a reduced flow rate compared with the GDVN injector, so that the crystals are used more efficiently. Using LCP as a carrier medium thus greatly reduces the amount of protein required to collect a complete set of diffraction data.

Apart from membrane proteins, which can be crystallized in LCP and are amenable to LCP–SFX, there are many important soluble macromolecules that represent challenging crystallization targets (Garman, 2014 ▸). These include large protein complexes, protein–DNA and protein–RNA complexes and proteins with dynamic domains. Quite often, limited quantities of sub-10 µm crystals of such macromolecules are available; however, obtaining large crystals suitable for data collection at synchrotrons or producing large quantities of microcrystals for SFX using a liquid injector may represent formidable obstacles. Here, we have modified our LCP–SFX protocol to demonstrate that LCP can serve as a suitable medium for the efficient delivery of soluble protein crystals for crystallo­graphic data collection at XFELs.

## 2. Methods   

### 2.1. Lysozyme microcrystal sample   

Chicken egg-white lysozyme powder (Sigma; catalog No. 62970) was dissolved in 0.02 *M* sodium acetate pH 4.6 buffer to a final concentration of 50 mg ml^−1^. Crystallization was initiated by injecting 20 µl lysozyme solution (50 mg ml^−1^) into 1 ml precipitant solution [20%(*w*/*v*) NaCl, 6%(*v*/*v*) PEG 6000, 1 *M* sodium acetate pH 3.0] at room temperature. Lysozyme microcrystals with average dimensions of 5 × 2 × 2 µm formed immediately upon vortexing the resulting solution.

The suspension of lysozyme microcrystals was centrifuged at 500*g* for 5 min. The supernatant (precipitant; around 980 µl) was carefully removed and the remaining 40 µl of lysozyme microcrystal/precipitant solution was used for LCP preparations.

Finally, the concentrated suspension of lysozyme crystals (lysozyme concentration of ∼25 mg ml^−1^) was mixed with lipids (1:1 9.9 MAG:7.9 MAG by weight) in a volume ratio of 45:55 using a dual-syringe lipid mixer until a homogeneous LCP formed (Cheng *et al.*, 1998 ▸; Caffrey & Cherezov, 2009 ▸).

### 2.2. Phycocyanin microcrystal sample   

*Thermosynechococcus elongatus* cells were preprocessed with a microfluidizer to break the cell walls. This was followed by a series of centrifugation cycles to isolate the thylakoid membrane. Phycobiliproteins such as phycocyanin (PC) and allophycocyanin (APC) were isolated by ultracentrifugation of the supernatant obtained after microfluidizer treatment. Cell debris and larger particles were spun down at 50 000*g* for 1 h. The supernatant was concentrated using Centricon spin filters with a molecular-weight cutoff of 100 kDa to obtain concentrated protein. PC microcrystals were produced by the free-interface diffusion method (Kupitz *et al.*, 2014 ▸) at a starting concentration of 50 mg ml^−1^ and using 75 m*M* HEPES pH 7, 20 m*M* MgCl_2_, 17% PEG 3350 as the precipitant. The final concentrations of protein and PEG 3350 were half of the starting values. The procedure of LCP sample preparation was identical to that described above. PC microcrystals with average dimensions of 10 × 10 × 5 µm were grown at 4°C over 9–14 h and pooled together before mixing with LCP as described above for the lysozyme samples.

### 2.3. XFEL data collection and treatment   

Experiments were performed using the CXI instrument (Boutet & Williams, 2010 ▸) at the Linac Coherent Light Source (LCLS) at SLAC National Accelerator Laboratory. LCLS was operated at a wavelength of 1.56 Å (7.95 keV), delivering individual X-ray pulses of nominally 35 fs pulse duration. Protein microcrystals in LCP medium were injected at an average flow rate of 170 nl min^−1^ into the XFEL beam focus region inside a vacuum chamber using an LCP injector with a 50 µm diameter nozzle. Single-shot diffraction patterns of randomly oriented crystals were recorded at 120 Hz with a Cornell–SLAC Pixel Array Detector (CSPAD) positioned at a distance of 100 mm from the sample (Hart *et al.*, 2012 ▸).

In the case of lysozyme, a total of 299 569 images were collected within 45 min, of which 119 844 were identified as crystal diffraction patterns by *Cheetah* (Barty *et al.*, 2014 ▸), corresponding to an average hit rate of 40%. In the case of PC, a total of 287 520 images were collected within 40 min, of which 18 794 were identified as crystal hits (average hit rate of 6.5%). The peak-detection parameters and the experimental geometry were optimized to ensure the best quality of peak finding and indexing. Autoindexing and structure-factor integration of the crystal hits was performed using *CrystFEL* (White *et al.*, 2012 ▸), which involved the application of fast Fourier transform (FFT)-based autoindexing algorithms, *MOSFLM* (Leslie, 2006 ▸), *DirAx* (Duisenberg, 1992 ▸) and *XDS* (Kabsch, 2010 ▸) followed by averaging and integration of Bragg peaks using a Monte Carlo algorithm. The final statistics of the quality of the data sets are summarized in Tables 1 ▸ and 2 ▸. The maximal radiation dose per crystal was estimated using *RADDOSE* (Paithankar *et al.*, 2009 ▸). σ(*I*) values were estimated as the standard deviations of the means of the intensity measurements (White *et al.*, 2012 ▸). The appropriate resolution cutoff was based on the behavior of the Pearson correlation coefficient CC_1/2_ *versus* resolution, and on the improvements in the *R*_work_/*R*_free_ values after including higher resolution shells in refinement (Karplus & Diederichs, 2012 ▸). After integration with *CrystFEL*, the initial phases were obtained by molecular replacement using known structures of the protein from the PDB (PDB entries 4et8 for lysozyme and 3l0f for PC; Boutet *et al.*, 2012 ▸; R. Fromme, D. Brune & P. Fromme, unpublished work) and the structures were refined using *phenix.refine* (Afonine *et al.*, 2012 ▸), including several simulated-annealing cycles in order to reduce phase bias. Structure images were prepared using *PyMOL* (Schrödinger). The final coordinates and structure factors were deposited in the PDB under accession codes 4zix (lysozyme) and 4ziz (PC).

## 3. Results   

**Figure 1:**
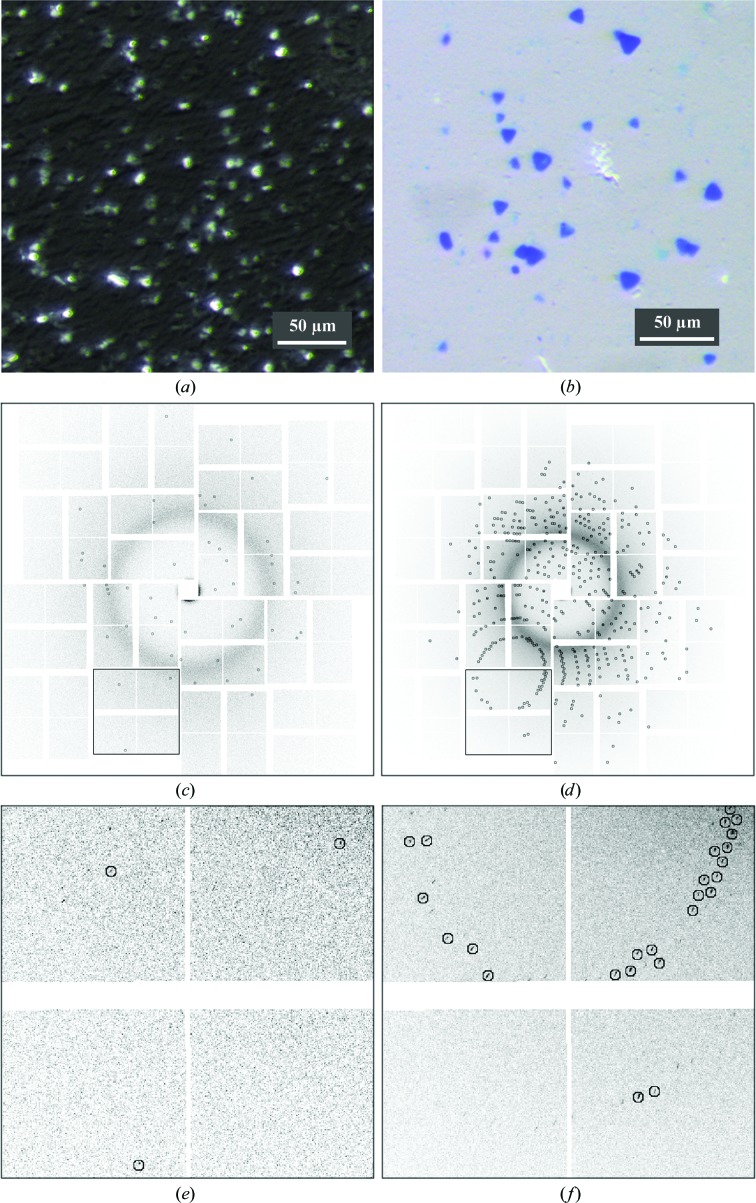
Representative crystal images and diffraction patterns. (*a*, *b*) Pictures of crystals embedded in LCP for lysozyme (*a*) and PC (*b*). (*c*, *d*) Full diffraction patterns for lysozyme (*c*) and PC (*d*). Parts of the diffraction images, outlined by squares in (*c*) and (*d*), are enlarged in (*e*) and (*f*), respectively, to facilitate visualization of individual diffraction peaks. Diffraction peaks identified by *Cheetah* are circled.

We have collected full XFEL diffraction data sets from two soluble proteins as model systems: the small, 14.3 kDa, chicken egg-white lysozyme and the relatively large, heterohexameric, 120 kDa, phycocyanin disk-like complex involved in light harvesting in photosynthesis as part of a phycobilisome in cyanobacteria. The proteins were first crystallized in their corresponding crystallization buffers, after which the slurries of microcrystals were concentrated by centrifugation to the desired concentration and mixed with appropriate LCP host lipids (Figs. 1 ▸ *a* and 1 ▸ *b*) using a lipid syringe mixer as described previously (Liu *et al.*, 2014 ▸). Mechanical mixing allows the fast and efficient formation of LCP; however, the shear forces can damage or dissolve the crystals if mixing is too vigorous. We observed that with gentle mixing, crystals over 10–20 µm in size, especially those with needle or plate-like shapes, tended to break into smaller pieces of less than 10 µm in size which, in turn, are sturdy enough to withstand the mechanical stress associated with mixing. Such crystal breakage did not affect the diffraction properties of the crystals in our test samples. The resulting dispersion of microcrystals in LCP was then transferred to an LCP injector and diffraction data (Figs. 1 ▸ *c* and 1 ▸ *d*) were collected at 120 Hz at the LCLS using an LCP flow rate of 170 nl min^−1^ as described previously (Liu *et al.*, 2013 ▸; Weierstall *et al.*, 2014 ▸).

### 3.1. Lysozyme structure   

**Figure 2:**
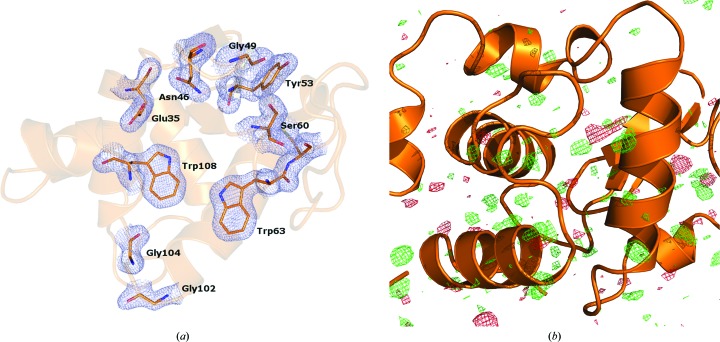
Lysozyme structure. (*a*) Overall structure of lysozyme showing 2*mF*_o_ − *DF*_c_ electron density around several residues in the active site contoured at 1σ. (*b*) Difference electron density between the data collected in this work and those presented in Boutet *et al.* (2012 ▸), contoured at 3σ. Positive difference density is green and negative is red.

The structure of lysozyme was solved at 1.9 Å resolution using 54 544 indexed single-crystal diffraction snapshots. The resulting overall structure is very similar to the previously published lysozyme structure determined by SFX using a GDVN injector (Boutet *et al.*, 2012 ▸). Since the software has substantially advanced over the last two years, we have also reprocessed the data from Boutet *et al.* (2012 ▸) using more recent versions of *Cheetah* (v.2013.3) and *CrystFEL* (v.0.5.3a), resulting in considerably improved data statistics. Both data sets were processed in the same way using the same versions of the software to avoid bias (Table 1 ▸). We compared the structures of lysozyme obtained using both LCP and GDVN injectors to ensure the validity of our procedure. The structures aligned very closely, with an r.m.s.d. of only 0.5 Å. The *B*-factor distributions are very similar, with a slightly higher average *B* factor in the case of the LCP structure. The quality of the maps is also very similar, with no structural differences observable in difference maps (Figs. 2 ▸ *a* and 2 ▸ *b*). Small differences were only detected around the side chains of solvent-exposed bulky amino-acid residues. These minor differences can be expected given the variations in the crystal-preparation protocols, crystal size and crystal-delivery methods. We can therefore conclude that using LCP as a carrier medium for SFX data collection results in a similar quality structure compared with the previously used GDVN injector, while offering the important advantage of consuming considerably less crystallized protein (0.1 mg using the LCP injector *versus* 15 mg using the GDVN injector).

### 3.2. Phycocyanin structure   

**Figure 3:**
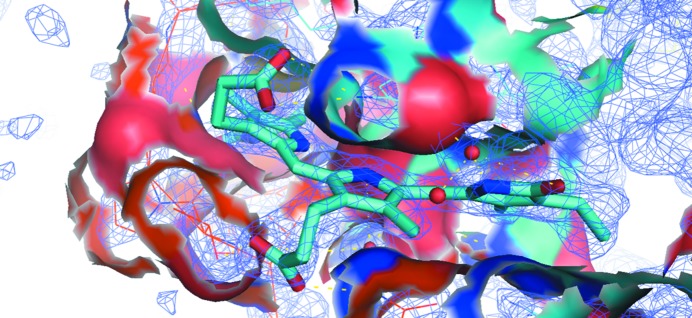
Phycocyanin structure. Simulated-annealing composite OMIT 2*mF*_o_ − *DF*_c_ electron-density map contoured at the 1.5σ level for the chromophore phycobilin inside its binding pocket. The map was built using 1.75 Å resolution XFEL data from PC crystals delivered in LCP.

As the second test protein, microcrystals of phycocyanin (PC), a photosynthetic pigment protein from the thermophilic cyanobacterium *T. elongatus*, were used. SFX data were collected using the LCP injector and compared with the structure of PC obtained previously using the GDVN injector. Both structures were of very similar resolution and quality. During the LCP–SFX experiment, data were collected from less than 7 µl of LCP sample (containing 3 µl crystal suspension), which yielded 18 794 hits, from which 6629 patterns were indexed at 1.75 Å resolution (Table 2 ▸). The quality of the structural model can be assessed from the details presented for one of the chromophores of PC shown in Fig. 3 ▸. The simulated-annealing composite OMIT 2*mF*_o_ − *DF*_c_ electron-density map at a contour level of 1.5σ fits tightly to the chromophore, which is difficult to achieve (even at higher resolution). Most importantly, using the LCP–SFX technique, the amount of protein crystals used is dramatically reduced, even though crystallization was carried out in liquid medium (as described in §[Sec sec2]2). Hence, previously established crystallization conditions can be successfully coupled with this technique. The entire data set required just 3 µl of crystal suspension (∼0.1 mg of protein), compared with the hundreds of microlitres of crystal suspension (∼30 mg of protein) that were used for the GDVN structure. The quality of the resulting electron-density map is very high, despite only 6629 crystals contributing to the data set.

## 4. Discussion   

Several considerations affect the use of LCP as a delivery matrix, the most important of which are the choice of the LCP host lipids and the optimal crystal size and density. The lipid choice is dictated by two factors. The first factor is the compatibility of the lipids with LCP extrusion in vacuum. The XFEL beam path usually runs in vacuum to reduce X-ray scattering. Previously, we have observed that the most commonly used lipid for LCP crystallization, monoolein, can partially solidify, forming the lamellar crystalline Lc phase. upon the injection of monoolein-based LCP in vacuum (Weierstall *et al.*, 2014 ▸). The Lc phase produces strong diffraction that can damage the detector; therefore, it should be avoided. Monoolein belongs to a lipid class known as monoacylglycerols (MAGs), which are composed of glycerol attached to a single fatty-acid chain through an ester bond. Monounsaturated MAGs used commonly for LCP applications are often referred to as *N*.*T* MAG based on the number of carbon atoms between the ester and the double bond (*N*) and between the double bond and the terminal methyl group (*T*). Therefore, monoolein corresponds to 9.9 MAG in this terminology. We have found that the shorter-chained monoolein analogues 9.7 MAG and 7.9 MAG, as well as a 1:1 mixture of 9.9 MAG and 7.9 MAG, do not form the Lc phase upon injection into vacuum and therefore are suitable as host lipids when LCP is extruded in vacuum. We should note that this limitation is relieved when LCP is extruded at atmos­pheric pressure, and all common LCP-forming lipids that were tested, including monoolein, can be used for this purpose.

The second factor is the compatibility of LCP with the precipitant solution used for protein crystallization. Many components of crystallization cocktails can disrupt LCP when used at high concentrations or extreme pH values (Cherezov *et al.*, 2001 ▸; Joseph *et al.*, 2011 ▸). Of all of the MAGs, monoolein-based LCP is one of the most stable towards the broadest range of commonly used precipitants. It is likely that new LCP-forming lipids with even higher stability will be identified. The compatibility of the chosen precipitant solution should be tested with different combinations of lipids before producing the actual samples containing protein crystals. Instability of LCP towards a particular precipitant composition can often be rectified by lowering the concentration of one of the components or by replacing one of the components by another, if this does not adversely affect the stability of the protein crystals. In the case of lysozyme, the precipitant was compatible with 7.9 MAG, 9.7 MAG and 9.9 MAG/7.9 MAG mixture lipids, while the PC precipitant was not compatible with 9.7 MAG but worked well with a 1:1 9.9 MAG:7.9 MAG mixture.

Data collected by SFX contain many randomly changing parameters, such as the exact crystal orientation, the crystal size and mosaicity and the XFEL pulse intensity and energy. Data processing relies on averaging of all these fluctuations, in the simplest case by Monte Carlo integration over many observations of the same reflection, and this requires a high multiplicity of data for convergence. Therefore, for efficient SFX data collection it is important to attain a high density of microcrystals, ensuring a high crystal hit rate. A clear advantage of working with crystals of soluble proteins, compared with membrane-protein crystals grown in LCP, is the ability to concentrate the crystal slurry to the desired concentration simply by centrifugation. Thus, the optimal concentration of crystals in LCP can be achieved, which corresponds to a 30–40% hit rate (number of images with crystal diffraction/total number of images). Higher hit rates would increase the occurrence of diffraction patterns from multiple crystals owing to the high density of crystals in the LCP stream, which is undesirable. Having adjusted the crystal density to an optimal value, we could collect full data sets in the shortest time: 43 min for lysozyme and 40 min for PC.

While this manuscript was under preparation, a paper describing soluble protein crystal delivery in a grease matrix using a different injector was published (Sugahara *et al.*, 2014 ▸). One of the principal differences between grease and LCP as the crystal-delivery matrix is that in LCP the crystals remain in contact with the original precipitant solution, while upon mixing with grease most of the precipitant solution is removed. We anticipate that different crystals could have different stabilities in such disparate matrices, and therefore further development of both of these and other approaches will expand the usage of SFX for challenging soluble protein targets at XFEL sources.

## Supplementary Material

PDB reference: lysozyme, 4zix

PDB reference: phycocyanin, 4ziz

## Figures and Tables

**Table 1:** XFEL data-collection and refinement statistics for lysozyme Values in parentheses are for the highest resolution shell.

	Lysozyme, LCP injector	Lysozyme, GDVN injector†, reprocessed
Data collection		
Average crystal dimensions (m)	2 2 5	1 1 2
Amount of protein used (mg)	0.1	15
Wavelength ()	1.56	1.32
Pulse duration (ns)	35	40
Beam size (m)	1.5	3
Flux (photons per pulse)	5 10^9^	4 10^11^
Maximum dose per crystal (MGy)	2.5	33
Space group	*P*4_3_2_1_2	*P*4_3_2_1_2
Unit-cell parameters ()	*a* = *b* = 79.1, *c* = 38.6	*a* = *b* = 78.9, *c* = 37.9
No. of collected images	299569	1512940
No. of hits/indexed images	119844/54544	69621/24465
Total/unique reflections	27923651/10266	10413069/10006
Resolution ()	27.971.89 (1.961.89)	35.261.90 (1.971.90)
Completeness (%)	100.0 (100.0)	100.0 (100.0)
Multiplicity	26558 (271.4)	10407 (933.9)
*I*/(*I*)	9.5 (1.9)	8.9 (4.3)
CC_1/2_	99.1 (68.1)	98.9 (93.7)
*R*_split_ (%)	8.2 (51.4)	8.9 (20.8)
Refinement		
No. of reflections	10266 (948)	9856 (892)
No. in test set	520 (53)	500 (52)
*R*_work_/*R*_free_ (%)	16.5 (25.2)/19.1 (25.1)	16.5 (16.1)/19.8 (24.7)
No. of atoms		
Protein	1015	1001
Water and others	93	90
*B* factors (^2^)		
Wilson/overall	28.6/29.8	24.3/25.3
Protein	29.0	24.50
Water and others	37.0	34.50
R.m.s.d., bonds ()	0.003	0.010
R.m.s.d., angles ()	0.71	1.2
Ramachandran plot statistics (%)		
Favored	99.2	99.0
Allowed	0.8	1.0
Disallowed	0	0

† Data from Boutet *et al.* (2012 ▸).

**Table 2:** XFEL data-collection and refinement statistics for PC Values in parentheses are for the highest resolution shell.

	PC, macrocrystal (PDB entry 3l0f)	PC, GDVN injector	PC, LCP injector
Data collection			
Average crystal dimensions (m)	200 200 300	10 10 5	10 10 5
Amount of protein used (mg)	N/A	30	0.1
Wavelength ()	1.00	1.45	1.56
Maximum dose per crystal (MGy)	N/A	N/A	66
Space group	*H*32	*H*32	*H*32
Resolution ()	19.01.35 (1.421.35)	36.41.95 (2.021.95)	31.61.75 (1.811.75)
Unit-cell parameters ()	*a* = *b* = 187.1, *c* = 59.8	*a* = *b* = 186.4, *c* = 60.3	*a* = *b* = 187.1, *c* = 60.5
No. of hits/indexed images	N/A	36118/16689	18794/6629
Total/unique reflections	600252/86960	7520260/32291	6171418/44284
*I*/(*I*)	19.15 (2.35)	2.92 (1.44)	2.94 (1.33)
Multiplicity	7.0 (5.9)	358.6 (47.9)	139.4 (36.8)
Completeness (%)	99.9 (99.7)	99.98 (99.8)	99.97 (100)
CC_1/2_	0.999 (0.799)	0.975 (0.34)	0.951 (0.32)
*R*_split_ (%)	NA	31.5 (97.3)	39.1 (94.2)
*R*_merge_ (%)	7.7 (84.9)	N/A	N/A
Refinement			
No. of reflections	82594 (6097)	32286 (3188)	40257 (4000)
No. in test set	4367 (296)	1660 (176)	1838 (183)
*R*_work_/*R*_free_ (%)	13.6 (27.1)/17.5 (33.1)	24.0 (37.5)/28.7 (41.9)	20.4 (46.9)/25.2 (50.2)
No. of atoms			
Protein	2497	2497	2497
Water and others	573	258	300
*B* factors (^2^)			
Wilson/overall	16.3/16.5	15.3/24.1	33.8/37.5
Protein	20.7	23.9	37.0
Water and others	41.5	27.8	45.6
R.m.s.d., bonds ()	0.007	0.024	0.012
R.m.s.d., angles ()	1.58	1.29	1.24
Ramachandran plot statistics (%)			
Favored	98.2	98.2	98.5
Allowed	1.8	1.8	1.5
Disallowed	0	0	0
